# The prevalence and phenotypic manifestations of polycystic ovary syndrome (PCOS) among infertile Sudanese women: a cross-sectional study

**DOI:** 10.1186/s12905-022-01762-6

**Published:** 2022-05-13

**Authors:** Alawia N. Elasam, Mohamed A. Ahmed, Abdel B. A. Ahmed, Manal E. Sharif, Abdalla Abusham, Bahaeldin Hassan, Ishag Adam

**Affiliations:** 1 Faculty of Medicine, University of Khartoum, Khartoum, Sudan; 2 College of Medicine, King Khalid University, Abha, Saudi Arabia; 3 Department of Obstetrics and Gynecology, Unaizah College of Medicine and Medical Sciences, Qassim University, Unaizah, 56219 Saudi Arabia

**Keywords:** Phenotype, Polycystic ovary syndrome (PCOS), Infertility, Manifestations

## Abstract

**Background:**

Polycystic ovary syndrome (PCOS) is a global health problem associated with significant morbidity during reproductive age. Only a few published studies that address the clinical manifestations and phenotypic presentation of the disease have been conducted in Africa, including Sudan. Thus, this study aimed to evaluate the clinical and biochemical presentation of the different PCOS phenotypes among infertile Sudanese women.

**Methods:**

A cross-sectional, descriptive study was conducted from January to December 2019. A total of 368 infertile women with PCOS (based on the Rotterdam criteria) were recruited from a fertility center in Khartoum, Sudan. Clinical, hormonal, and ultrasonographic characteristics were described and compared between the four phenotypes of PCOS.

**Results:**

Majority (321 [87.2%]) of the women had oligo/anovulation (OA). Polycystic ovary morphology on ultrasound appeared in 236 (64.1%) women, acne in 171 (46.5%) women, acanthosis nigricans in 81 (22.0%) women, and hirsutism in 101 (27.4%) women. Phenotype D was the most prevalent among infertile Sudanese women (51.6%), followed by phenotype B (22.6%), phenotype C (18.2%), and phenotype A (7.6%). No statistical differences in the body mass index and hormonal profile between the four phenotypes were noted. Women with phenotype A were older and had high mean blood pressure, and a higher waist/hip ratio was observed among women with phenotype D.

**Conclusion:**

Unlike the global distribution of PCOS phenotypes, Sudanese women uniquely expressed phenotype D as the most prevalent. More epidemiological studies are needed in the region due to geographical, ethnic, and genetic variations.

## Plain English summary

Polycystic ovary syndrome (PCOS) is a global health problem associated with significant drawbacks during reproductive life. Few published studies have been conducted in Africa (including Sudan) addressing the clinical manifestations and phenotypic presentation of the disease. Therefore, we aimed to evaluate the clinical and biochemical presentation of the different PCOS phenotypes among infertile Sudanese women. A total of 368 infertile women with PCOS from a fertility center in Khartoum, Sudan, participated in the study. Clinical, hormonal, and ultrasonographic characteristics were described and compared between the four phenotype groups of PCOS. In this regard, Sudanese women uniquely expressed phenotype D as the most prevalent, and this does not match with the global distribution of PCOS phenotypes. Moreover, women with phenotype A were older and had high mean blood pressure, and a higher waist/hip ratio was observed among women with phenotype D. More epidemiological studies on this subject are needed in the region due to geographical, ethnic, and genetic variations.

## Background

Polycystic ovary syndrome (PCOS) is a combination of metabolic, endocrinological, and genetic disorders. This syndrome is common during the reproductive age, with a global prevalence of 5–20% [1, 2]. PCOS can lead to poor obstetric outcomes such as infertility, pregnancy loss, gestational diabetes, and macrosomia [1]. Furthermore, metabolic syndrome, insulin resistance, hypertension, dyslipidemia, and increased cardiovascular disease risk are recognized complications of this condition [3, 4]. Accumulating evidence suggests that one of the most important mechanisms of PCOS pathogenesis is insulin resistance [3, 4]. For this reason, the use of insulin sensitizers, such as inositol isoforms, has gained increasing attention due to their safety profile and effectiveness [5, 6].

The European Society for Human Reproduction and Embryology (ESHRE) and the American Society of Reproductive Medicine (ASRM) have set criteria for the diagnosis of PCOS (Rotterdam 2003) [7]. The Rotterdam criteria include oligo-anovulation (OA) (cycles greater than 35-day intervals or 8 cycles or less per year), hyperandrogenism (HA), and polycystic ovary morphology (PCOM) (ovarian volume of 10 ml and/or an antral follicle count [AFC] of more than 12 cysts of 2–9 mm in diameter, any two being enough for the diagnosis of PCOS) [7]. Based on Rotterdam criteria, four main phenotypes were described: phenotype A, composed of OA, HA, and PCOM; phenotype B (OA and HA); phenotype C (HA and PCO); and phenotype D (OA and PCO) [8]. ESHRE 2018 guidelines recommend the assessment of clinical HA (hirsutism, acne, and alopecia). In this regard, hirsutism was assessed through a modified Ferriman-Gallwey score (mFG) with a diagnostic level of ≥ 4–6 [9]. It is noteworthy that the expression of PCOS has varied depending on geographical and ethnic differences even in the same country [10]. Furthermore, phenotype A has been reported as dominant in more than half of published clinical data [11, 12]. Even though data on PCOS in different settings have been published, no data on the epidemiology of PCOS in Africa exist [1]. Thus, we aimed to assess the clinical manifestations of PCOS phenotypes among infertile Sudanese women.

## Methods

### Study design and settings

A cross-sectional study was conducted at Saad Abualila Infertility Center (Khartoum, Sudan) from the first of January to the end of December 2019. During the study, the Strengthening the Reporting of Observational Studies in Epidemiology (STROBE) Statement: Guidelines for Reporting Observational Studies were strictly followed [13].

### The outcome measures

The main outcome measures were prevalence and clinical, hormonal, and ultrasonographic features of PCOS phenotypes.

### Study population and sampling techniques

All confirmed PCOS cases (according to Rotterdam criteria) of reproductive age (18–45 years) who attended the infertility center of the Saad Abualila Hospital at the time of the study were included. Exclusion criteria included the following: women with diabetes mellitus, cardiovascular diseases, pregnancy, and endocrine diseases and women who received hormonal treatments in the last six months before the study.

The sample size was determined using the formula of calculating the sample size of frequency in a population. We hypothesized that phenotype D would be prevalent in 50% of the women with PCOS, as there was no available data among the population in the region. However, previous reports have demonstrated that 51.6% of women expressed phenotype D of PCOS [14]. The resulting sample size was 384 with a 95% level of confidence and a 5% margin of error.

### Data collection

After signing informed consent, all participants passed through three stages: questionnaire, physical examination, and ultrasonography. The questionnaire includes dermatological complaints (acanthosis nigricans), sociodemographic data, and medical, obstetric, menstrual, and gynecological history. Physical examination was conducted by the gynecologist who ran the clinics. Weight in kilograms, height in centimeters, and blood pressure were measured. Waist/hip ratio (WHR) was measured according to World Health Organization (WHO) protocol using a stretch-resistant tape [15]. Hirsutism was assessed through the modified Ferriman-Gallwey (mFG) score. The presence of acne, acanthosis nigricans, and alopecia was detected through visual assessment as well. Ultrasonographic examinations were performed by for all participants; the number of follicles in each ovary and the ovarian volume were determined. A total of 5 ml of blood was drawn from each respondent to test follicle-stimulating hormone (FSH), luteinizing hormone (LH), and total testosterone (TT). All serum hormone analyses were conducted using an enzyme-linked immunosorbent assay (Stat Fax 2600 ELISA System, USA). Afterward, PCOS cases were identified and classified into the four phenotypes: A, B, C, and D.

### Definitions and measurements

PCOS diagnosis was made in the presence of at least two criteria: OA defined as cycles (vaginal bleeding episodes) greater than 35–day intervals or 8 cycles or less per year and biochemical and/or clinical HA**.** The hirsutism is defined at the cut-off of ⩾ 4–6 mFG score as per ESHRE PCOS guideline 2018 [9]. PCO was detected under the guide of the Rotterdam criterion (ovarian volume of 10 ml and/or an AFC of 12 cysts or more, measuring 2–9 mm in diameter) [7]. The ultrasonography was operated by a gynecologist who ran the clinics and diagnosed PCOS cases. A transducer of a 6.5 MHz frequency was used for this purpose. To measure biochemical HA, TT was measured using an immunoassay analyzer (AIA 360, Tosoh, Japan), and levels > 0.88 ng/mL were considered diagnostic for HA [16]. BMI was calculated as weight in kilograms divided by the squared height in meters. WHO classification was used to classify the women according to their BMIs as follows: normal weight (18.5–24.9 kg/m^2^), overweight (25.0–29.9 kg/m^2^), or obese (30.0–34.9 kg/m^2^) [17]. Blood pressures > 140 systolic and > 90 diastolic were considered abnormal. Hypertension defined as blood pressure > 140/90 mmHg was noted on two or more occasions using the same calibrated mercury manometer.

Furthermore, according to WHO protocol, the waist circumference was measured at the midpoint between the lower margin of the last palpable ribs and the top of the iliac crest using stretch‐resistant tape. Hip circumference was measured around the widest portion of the buttocks, with the tape parallel to the floor. PCOS cases were divided into four phenotypes: A (OA, HA, and PCO), B (HA and OA), C (HA and PCO), and D (OA and PCO).

### Statistical analysis

Data were entered into a computer using SPSS for analysis. Continuous data were assessed for normal distribution using the Shapiro–Wilk test. All continuous data were not found to be normally distributed and were compared between the four phenotype groups of PCOS using Kruskal–Wallis. Dichotomous variables were compared with a two-tailed Chi-square or Fischer exact test where appropriate. *P*-value > 0.05 was considered statistically significant.

## Results

Out of 384 PCOS women who were targeted as our sample size, 368 were enrolled in the study after the exclusion of 16 subjects who did not fulfill the inclusion criteria.

The median (interquartile range) age of the sample was 26.0 (7.9) years; moreover, 274 (71.7%) women have higher than a secondary level of education, and 156 (42.4%) were employees (Table 1). Physical examination revealed that 156 (42.4%) women were obese and 10 (2.7%) had hypertension (Table 1).

**Table 1:** Sociodemographic and clinical characteristics of infertile women in Khartoum, Sudan

Variables	Number	Frequency
Age, years*	26.0	7.9
*Education level*		
≥ Secondary level	264	71.7
< Secondary level	104	28.3
*Occupation*		
Housewives	212	57.6
Employee	156	42.4
*Body mass index (kg/m*^*2*^*)*		
Underweight	12	3.3
Normal weight	98	26.6
Overweight	102	27.7
Obese	156	42.4
*Oligo/Anovulation*		
No	47	12.8
Yes	321	87.2
*Hirsutism (modified Ferriman Gallwey score)*		
(mF-G) ⩾6	101	27.4
(mF-G) < 6	267	72.6
*Ultrasonographic features*		
PCO morphology	236	64.1
Normal ovaries	132	35.9
*Acne*		
Present	171	46.5
Absent	197	53.5
*Acanthosis Nigricans*		
Present	81	22.0
Absent	287	78.0
*Blood pressure*		
Hypertensive	10	2.7
Normotensive	358	97.3

*The median (interquartile range)

**Fig. 1:**
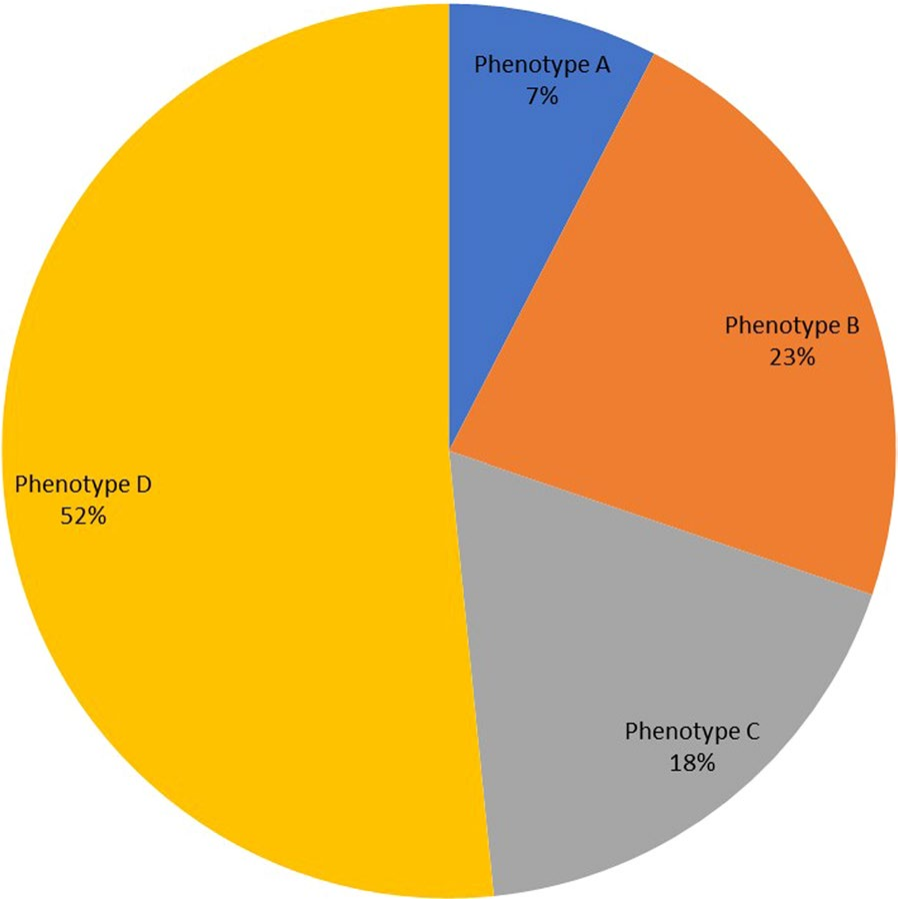
Frequency of PCOS phenotypes in Khartoum, Sudan

The clinical presentation of infertile Sudanese women is shown in Table 1. The median (interquartile range) of the WHR was 0.97 (0.05) cm. The majority of the women (321 [87.2%]) presented OA. PCO appeared in 236 (64.1%), acne in 171 (46.5%), acanthosis nigricans in 81 (22.0%), and hirsutism in 101 (27.4%) (Table 1). Phenotype D was the most prevalent among infertile Sudanese women (51.6%), followed by phenotype B (22.6%), phenotype C (18.2%), and phenotype A (7.6%) (Table 2, Fig. 1).

**Table 2:** Clinical manifestations and frequency of polycystic ovary syndrome phenotypes in Khartoum, Sudan

Characteristics	Phenotypes			
	A (N = 28)	B (N = 83)	C (N = 67)	D (N = 190)
OA	Present	Present	Absent	Present
HA	Present	Present	Present	Absent
PCO	Present	Absent	Present	Present
Frequency (%)	7.6	22.6	18.2	51.6

Furthermore, no statistical differences in BMI, hormonal profile (TT, LH, FSH), and LH/FSH ratio were detected between the four phenotypes (Table 3). Significantly higher median age and mean blood pressure were reported in women with phenotype A when compared with other phenotypes: (*P* = 0.046) and (*P* = 0.001), respectively. WHR was significantly higher among women with phenotype D (*P* = 0.043) compared to phenotype groups A, B, and C (Table 3).

**Table 3:** Clinical, hormonal and participant’s characteristics among the four polycystic ovary syndrome phenotypes in Khartoum, Sudan

Variables	Phenotype A	Phenotype B	Phenotype C	Phenotype D	*P*
Age, years	27.5 (9.5)	25.0 (9.0)	26.0 (9.0)	26.0 (6.0)	0.046
Body mass index, kg/m^2^	28.4 (8.3)	27.5 (6.6)	29.5 (6.4)	29.6 (7.5)	0.093
Waist/hip ratio	0.97 (0.08)	0.97 (0)	0.97 (0.09)	0.97 (0.46)	0.043
Mean blood pressure, mmHg	63.8 (0.1)	63.8 (0)	63.8 (0)	63.8 (0)	0.001
Luteinizing hormone, IU/L	7.82 (1.7)	7.82 (1.8)	7.82 (0.8)	7.32 (3.9)	0.777
Follicle-stimulating hormone, IU/L	3.8 (1.7)	4.2 (4.1)	4.2 (2.3)	3.2 (2.3)	0.337
Luteinizing hormone/ follicle-stimulating hormone	2.05 (0.72)	1.68 (0.82)	1.68 (0.73)	2.28 (0.9)	0.885
Total testosterone, ng/dL	28.4 (0)	28.4 (0)	28.4 (0)	28.4 (0)	0.117

## Discussion

In the present study, 42.4% of the women were obese. Another published study demonstrated that BMI was significantly higher among infertile PCOS cases in Khartoum, Sudan [18]. A higher frequency (63.7%) of obesity was also noted in PCOS women in California, USA [19]. However, a lower prevalence of obesity (31.4%) was reported among infertile Jordanian women with PCOS [20]. In the current study, 87.2% of the women with PCOS had OA, and this aligns with the previous finding that OA accounts for 70% of the cases [21]. However, Azziz et al. observed that approximately 80% of the women with PCOS had OD [22].

Approximately two-thirds of the women in this study presented diagnostic ultrasonographic features of PCO. Likewise, it has been documented that PCOM on ultrasound is the most common feature of PCOS [12]. This finding was comparable to a previous study from Denmark [23], lower than findings from China (94.2%) [24], and higher than the prevalence reported among Tanzanian women (78.1%) [25]. Perhaps the low predictive value of PCOM could be explained by whether 5 MHz or 7.9 MHz ultrasound probes were used [12, 23]. Clinical HA is widely manifested among PCOS patients. In this regard, Jamil et al. have reported that most of the investigated women with PCOS had HA [26]. In our study, we observed that 27.4% of participants had hirsutism. Similar results of hirsutism (30.6%) were demonstrated in an infertile cohort of Nigerian women with PCOS [27]. The prevalence of hirsutism was nearly double (60.4%) among infertile women with PCOS in Egypt [28]. Ultimately, the expression of hirsutism varies depending on the geographical regions, ethnicity, and genetics, and it is important to develop a population-specific cut-off level. Approximately 70% of women with hirsutism have PCOS [29].

In the current study, approximately half (46.5%) of the women had acne. A much lower incidence (15.6%) of acne was reported in China [24]. These wide variations could be attributed to the ethnic differences between Asian and African populations. Around a fifth (22.0%) of the women in our study showed acanthosis nigricans at the time of examination. This cutaneous manifestation was similarly documented in Erbil, Iraq (28.5%) [26]. Another study among Vietnamese women with PCOS indicates that only 1.5% exhibited acanthosis nigricans [16]. These differences could be explained by the nature of the study’s population. Contrary to the Vietnamese study, our study and the Iraqi study were conducted on infertile cohorts.

On another note, in the current study, phenotype D was the most prevalent among infertile Sudanese women (51.6%), followed by phenotype B (22.6%), phenotype C (18.2%), and phenotype A (7.6%). These findings align with a study from China and Iran [14] that reported phenotype D as the most prevalent. Furthermore, we observed unique distribution among infertile Sudanese women indicating phenotype A as the least prevalent (7.6%). Globally published data indicate that more than half of PCOS patients identified within the clinical settings demonstrate phenotype [11, 12]. Overall, it seems that the classic form of PCOS (i.e., phenotypes A and B) constitutes approximately two-thirds of the total of PCOS patients identified within the clinical settings [30]. Unfortunately, insufficient data exist regarding the distribution of phenotypes in women with PCOS identified in medically unbiased (i.e., unselected) populations, which would more accurately reflect the distribution of phenotypes in PCOS in the “natural” state. A meta-analysis of studies on PCOS has provided further evidence of referral bias for the PCOS phenotype [11].

Few published studies recently documented the distribution of PCOS phenotypes using Rotterdam criteria [31, 32]. In these studies, around two-thirds of PCOS women expressed phenotypes B and C; however, phenotype A and phenotype D had equal rates of prevalence. It is noteworthy that the differences in the rates of expression of PCOS phenotypes even in the same settings could be explained by whether the assessment of the women occurred either while they were seeking medical care or while they were attending a routine health assessment [19, 33].

In this study, no statistical differences in BMI, hormonal profile (TT, LH, FSH), and LH/FSH ratio were observed between the four phenotypes. However, Jamil et al. observed higher BMI among women with phenotypes A and B, but they agreed on the absence of significant differences in the hormonal profile (LH and FSH) [26]. In line with our findings, Sahmay et al. did not report significant differences in TT among the four phenotypes [34].

In this study, phenotype A women were significantly older and had higher blood pressure than women with other phenotypes. The association between advanced age and increased blood pressure among PCOS women was confirmed in a recent Brazilian study [35]. Furthermore, it has been reported that the classic PCOS (phenotypes A and B) can be referred to as the complete phenotype [36].

Additionally, the results of the current study revealed that WHR was significantly higher among women with phenotype D compared to women with phenotypes A, B, and C. In contrast to our findings, other studies reported that WHR was higher in the classic phenotypes A and B [16, 37]. WHR reflects truncal obesity, insulin resistance, and metabolic syndrome. However, various previous studies did not report a significant difference in insulin resistance and dyslipidemia between different PCOS phenotypes [38]. An assessment of studies in Africa highlights the lack of research among Black women in Sub-Saharan Africa, where no large-scale epidemiologic studies of PCOS have been conducted and only very few studies on the PCOS phenotype exist [25, 27, 39]. Furthermore, PCOS among Black women may be associated with additional or more severe morbidities, such as uterine leiomyomata, compared to White women [40]. Furthermore, future research must address several points such as current challenges for ovarian stimulation in patients affected by PCOS [41, 42].

## Conclusion

Infertile Sudanese women uniquely expressed phenotype D as the most prevalent PCOS phenotype. The majority of the study population presented OA, followed by PCOS and clinical HA. No published data on PCOS phenotypes in Sudan have been published, and only a few studies have been conducted in Africa. Therefore, this highlights a need for epidemiological studies in Africa concerning PCOS phenotypes, particularly due to genetic, racial, and geographical variations.

## Data Availability

The datasets used and/or analyzed during the current study are available from the corresponding author on reasonable request.

## Abbreviations

PCOS: Polycystic ovary syndrome
ESHRE: The European Society for Human Reproduction and Embryology
ASRM: The American Society of Reproductive Medicine
AFC: Antral follicle count
OA: Oligo-anovulation
HA: Hyperandrogenism
PCOM: Polycystic ovary morphology
mFG: Modified Ferriman-Gallwey score
WHR: Waist/hip ratio
WHO: World Health Organization
FSH: Follicular stimulating hormone
LH: Luteinizing hormone
TT: Total testosterone
BMI: Body mass index
